# High Thyroglobulin Antibody Levels Increase the Risk of Differentiated Thyroid Carcinoma

**DOI:** 10.1155/2015/648670

**Published:** 2015-10-27

**Authors:** Jing Qin, Zhenqian Yu, Haixia Guan, Liangfeng Shi, Yongping Liu, Na Zhao, Zhongyan Shan, Cheng Han, Yushu Li, Weiping Teng

**Affiliations:** ^1^Department of Endocrinology and Metabolism, Institute of Endocrinology, Liaoning Provincial Key Laboratory of Endocrine Diseases, The First Affiliated Hospital of China Medical University, No. 155 Nanjing North Street, Shenyang, Liaoning 110001, China; ^2^Department of Endocrinology and Metabolism, Jinshan Hospital, Fudan University, No. 391 Jinyi Road, Jinshan District, Shanghai 201508, China

## Abstract

*Background*. Despite the many studies examining thyroid cancers, the effect of thyroid autoantibodies on differentiated thyroid carcinoma (DTC) remains unclear. *Objective*. To investigate the association between serologic thyroid autoantibodies (ATAs) and DTC, we retrospectively evaluated data of thyroid nodules obtained from patients who underwent thyroid surgery. *Methods*. Data of thyroid nodules obtained from 1,638 patients who underwent thyroid surgery were evaluated. Thyroid autoimmunity was assessed by the presence of thyroglobulin (TgAb) or thyroid peroxidase antibodies (TPOAb). *Results*. Among our study cohort, the prevalence of elevated TgAb (≥40 IU/mL) and TPOAb (≥50 IU/mL) was higher in patients with DTC than those with benign nodules. Patients with DTC and elevated TgAb had a higher prevalence of extrathyroidal invasion. In the multivariate analysis, TgAb ≥ 40 IU/mL was significantly associated with DTC (odds ratio [OR] = 2.10, 95% confidence interval [CI] 1.40–3.15) compared with TgAb < 40 IU/mL group, independent of other confounding factors such as decreased age, single nodule, and elevated TSH level. In conclusion, elevated TgAb was associated with DTC. *Conclusions*. This study revealed that high levels of TgAb may act as an independent prediction factor for DTC, and suggests that patients with high TgAb concentrations may be predisposed to DTC.

## 1. Introduction

Although thyroid carcinoma only accounts for approximately 1% of all cancers, many epidemiologic studies have revealed that thyroid carcinoma is the most common head and neck malignancy worldwide, including China [1–3]. The majority of thyroid cancers are either papillary thyroid carcinoma (PTC) or follicular thyroid carcinoma (FTC), which together represent more than 90% of all thyroid malignancies. Often, PTC and FTC are grouped together and referred to as differentiated thyroid carcinoma (DTC) [2, 4, 5].

In recent years, the incidence of thyroid cancer has been rising [1, 6]. Fine-needle aspiration biopsy (FNAB) is the “gold standard” for evaluating thyroid nodules, but the majority of nodules with FNAB results are classified as indeterminate [7]. Thus, the predictors that improve preoperative risk stratification for nodules are still needed. Some clinical parameters have been confirmed to be associated with DTC, such as young (<20) or old (>70) age, male gender, history of ionizing or exposure to radiation of the head and neck as a youth, family history of thyroid cancer, nodule size and type, or rapid growth associated with hoarseness [8–10]. In addition, many retrospective studies have linked higher serum TSH level with an increased risk of DTC [3, 11, 12]. Although these potential predictors may play an important role in the preoperative diagnosis of thyroid carcinoma, the need for other prediction factors exists.

The relationship between thyroid autoimmunity and DTC has been discussed for many years. However, data specifically correlating thyroid autoantibodies and DTC occurrence is limited. A few studies [13–20] have attempted to define this relationship, but the conclusions are debatable. Due to the controversy, as well as the high prevalence of DTC, we retrospectively evaluated the surgical data obtained from a large number of patients with thyroid nodules to assess the association between thyroid autoantibodies (ATAs) and DTC.

## 2. Materials and Methods

### 2.1. Patients

Between January 2011 and December 2013, the First Affiliated Hospital of China Medical University recruited 2,008 patients with thyroid nodules undergoing thyroid surgery. Physical examination, thyroid ultrasonography, and thyroid profiles were performed before surgical procedure. The data were analyzed retrospectively. In an effort to minimize potential factors affecting the reliability of this study, we exclude 156 patients (7.8% of 2,008 patients) who had previously been exposed to high levels of radiation, 60 patients (3%) whose final pathology revealed the presence of undifferentiated thyroid cancer, 80 patients (4%) who reported a history of surgery, and 74 patients (3.6%) of whom serum thyroid peroxidase antibody (TPOAb) or thyroglobulin antibody (TgAb) data were unavailable. The remaining 1,638 individuals (81.6% of 2,008 patients, 237 with DTC and 1,401 with benign results) were included in the study cohort.

### 2.2. Thyroid Profiles

Thyroid profiles, including serums TSH, TPOAb, and TgAb levels at a fasting state, were measured prior to thyroidectomy at the endocrine laboratory of the First Affiliated Hospital of China Medical University. Serums TSH, TgAb, and TPOAb levels were detected using the Abbott Architect (Abbott Laboratories, Abbott Park, IL, USA) with manufacturer's functional sensitivities (FS) of 0.01 mIU/L, 0.31 IU/mL, and 0.50 IU/mL, respectively. For TSH, the reference range (RR) was 0.3–4.8 mIU/L and the intra-assay and interassay coefficients of variation (CV) were 4.9% and 5.2%, respectively [21]. For TgAb and TPOAb, the intra-assay and interassay CV were less than 8.5%, and the RR were 0.00–4.11 IU/mL and 0.00–5.61 IU/mL, respectively. In this study, we use RR as cutoff of positive ATAs as it has been reported that RR was more appropriate for diagnosing thyroid autoimmunity and minimised false positive [22]. According to the experience in our previous prospective epidemiological studies [23, 24], we used 40 IU/mL, 100 IU/mL, and 500 IU/mL for further categorizing elevated TgAb and 50 IU/mL, 100 IU/mL, and 500 IU/mL for elevated TPOAb.

### 2.3. Thyroid Ultrasound

To characterize the thyroid nodule size, number, and echo pattern, thyroid nodules were evaluated preoperatively by four trained radiologists via high-resolution ultrasound images (Aplio80, TOSHIBA, Japan). A double-blinded test was designed to minimize potential intra- and interobserver discrepancies. Radiologist agreement on nodule number and abnormal echo pattern was almost 100% and intra- and interobserver CVs were 8.6% and 9.2%, respectively.

### 2.4. Histopathological Diagnosis

Final histological data were available for all the patients included in the cohort. An experienced pathologist assessed the presence or absence of DTC, including PTC and FTC. For patients with DTC, tumors were staged according to the system established by the American Joint Committee on Cancer with regard to tumor size, status of extrathyroidal invasion, lymph node metastasis, and distant metastasis.

### 2.5. Statistical Analysis

All continuous data were expressed as mean ± standard deviation (SD). Two continuous numeric variables with a normal distribution were analyzed using an independent Student's *t*-test. Comparison of continuous variables with nonnormal distribution was expressed as median and empirical percentiles and analyzed by a Mann-Whitney *U*-test. The chi-square test (*χ* ^2^) or Fisher's exact test were used for categorical variables. Finally, a binary logistic regression analysis was performed to evaluate the independent influence of factors. *P* values <0.05 were considered statistically significant. All statistical analyses were performed using SPSS software (version 16, Chicago, IL, USA).

### 2.6. Ethical Aspects

Informed consents were obtained from all the patients, and the Medical Ethics Committee of China Medical University approved all protocols.

## 3. Results

The clinical features of the study cohort are outlined in Table 1. The 1,638 patients consisted of 355 (21.7%) males and 1,283 (78.3%) females. Of them, 1,401 patients (85.5%) were diagnosed with benign tumors, and the remaining 237 patients (14.5%) were diagnosed with DTC, which was confirmed by histopathology. PTC accounted for the vast majority of the DTC diagnoses (96.6%, *n* = 229), and there was no difference in the sex ratio between patients with benign tumors and patients with DTC. Patients with DTC were relatively younger (43.4 ± 12.9 versus 48.4 ± 11.1 yr; *P* < 0.001) and had higher preoperative median TSH levels (1.53 mIU/L versus 1.05 mIU/L; *P* < 0.001) than those with benign nodules. In addition, the mean nodule size was smaller in patients with DTC compared to individuals without malignancy (2.7 ± 1.9 cm versus 3.3 ± 1.6 cm; *P* < 0.001). Moreover, solitary nodules were more common in patients with DTC (80/237, 33.8%) compared to patients with benign nodules (241/1401, 17.2%; *P* < 0.001). These data suggest a higher risk of DTC in individuals with a solitary nodule. We also found that patients with TgAb concentration more than 40 IU/mL were more common in DTC group than in benign nodule group (23.6% versus 12.6%, *P* < 0.001), the same with TPOAb concentration more than 50 IU/mL (12.7% versus 7.6%, *P* = 0.009), suggesting that elevated ATAs may associate with DTC.

**Figure 1:**
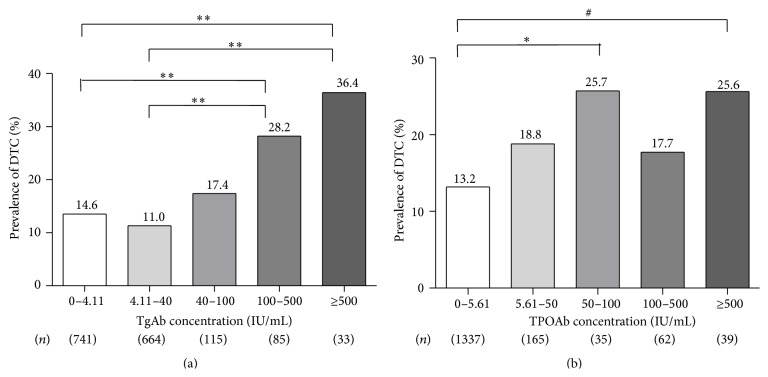
(a) Patients were subdivided into five TgAb concentration groups. The total number of patients (*n*) in each group is given beneath the graph. ^*∗∗*^ *P* < 0.005; *P* value for trend < 0.001. (b) Patients were subdivided into five TPOAb concentration groups. The total number of patients (*n*) in each group is given beneath the graph. ^*∗*^ *P* = 0.032; ^#^ *P* = 0.025; *P* value for trend = 0.015.

**Figure 2:**
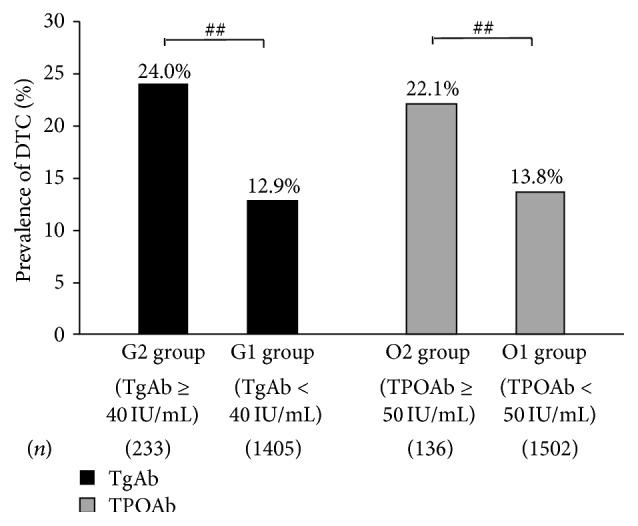
Prevalence of DTC according to the levels of TgAb or TPOAb concentration. The total number of patients (*n*) in each group is given beneath the graph. ^##^ *P* < 0.01. DTC: differentiated thyroid cancer.

Given the higher prevalence of elevated ATAs in patients with DTC, we next evaluated the rates of DTC according to different ATAs concentration levels. As shown in Figure 1, patients were divided into five category levels of TgAb or TPOAb concentrations. The prevalence of DTC corresponded with the increasing TgAb concentration and was significantly higher in the 100–500 IU/mL and ≥500 IU/mL groups than either negative group (28.2% versus 14.6% and 36.4% versus 14.6%, *P* < 0.005) or 4.11–40 IU/mL group (28.2% versus 11.0% and 36.4% versus 11.0%, *P* < 0.005) (Figure 1(a)). When we evaluated TgAb by dividing G1 group (which includes negative and 4.11–40 IU/mL groups, *n* = 1305) and G2 group (TgAb ≥ 40 IU/mL, *n* = 233), G2 group had a significantly higher DTC rate than G1 group (24.0% versus 12.9%, *P* < 0.01) (Figure 2). Similarly, the rate of DTC was significantly higher in the group of 50 ≤ TPOAb ≤ 100 IU/mL (25.7% versus 13.2%, *P* = 0.032) and TPOAb ≥ 500 IU/mL (25.6% versus 13.2%, *P* = 0.025) than in the negative TPOAb group (Figure 1(b)), and when we evaluated TPOAb by dividing O1 group (including negative and 5.61–50 IU/mL groups, *n* = 1502) and O2 group (TPOAb ≥ 50 IU/mL, *n* = 136), O2 group had significantly higher DTC prevalence than the O1 group (22.1% versus 13.8%, *P* < 0.01) (Figure 2). Overall, these data suggest that high levels of ATAs are associated with DTC.

Considering the connection between high levels of ATAs and DTC, we further evaluated various clinical parameters of DTC in two levels of ATAs concentration. As shown in Table 2, the 237 patients with DTC were divided into two TgAb groups (G1 group, TgAb < 40 IU/mL; G2 group, TgAb ≥ 40 IU/mL) or two TPOAb groups (O1 group, TPOAb < 50 IU/mL; O2 group, TPOAb ≥ 50 IU/mL). The mean age, mean nodule size, serum TSH levels, extrathyroidal invasion, and lymph node metastases were evaluated. Thirty patients (12.7%) were in O2 group, and 56 (23.6%) were in G2 group. Using this mode of characterization, the mean age of DTC was decreased in O2 group compared with O1 group (38.53 ± 13.50 versus 44.15 ± 12.64; *P* = 0.025), but no significant difference was observed in G2 group (41.23 ± 14.67 versus 44.13 ± 12.20; *P* = 0.184). In addition, there was no statistical significance in the mean nodule size between O2 group and O1 group (3.14 ± 0.55 cm versus 2.71 ± 0.14 cm; *P* = 0.95). This also held true for patients in G2 group and G1 group (3.02 ± 0.31 cm versus 2.49 ± 0.14 cm; *P* = 0.07). The median TSH level was significantly higher in O2 group (2.09 versus 1.46; *P* = 0.003), and a similar correlation was observed in G2 group (1.72 versus 1.44; *P* = 0.018). Moreover, the prevalence of extrathyroidal invasion significantly increased in patients of G2 group (30.4% versus 17.1%; *P* = 0.031), while no statistical significance was observed in O2 group (20% versus 20.3%; *P* = 0.971). Finally, there was no statistical significance in the prevalence of lymph node metastasis and advanced cancer stages between G2 group and G1 group (37.5% versus 28.7%, *P* = 0.214; 42.9% versus 36.5%, *P* = 0.389). This also held true for patients in O2 group (43.3% versus 29%, *P* = 0.112; 50% versus 36.2%, *P* = 0.146). Together, these data suggest that younger patients with high levels TgAb may have more severe DTC and extrathyroidal invasion.

We next sought to determine which factors might be independent risk predictors for DTC. To this end, binary logistic regression analysis (including gender, age, nodule type, serum TSH, TPOAb ≥ 50 IU/mL, and TgAb ≥ 40 IU/mL) was conducted, and the odds ratio (OR) in favor of having DTC was calculated. As shown in Table 3, the prevalence of DTC was significantly associated with TgAb ≥ 40 IU/mL (OR = 2.1, 95% confidence interval [CI] 1.40–3.15) compared with TgAb < 40 IU/mL group, but not with TPOAb ≥ 50 IU/mL (OR = 1.20, 95% CI 0.71–2.04).

As with TgAb, elevated TSH levels (OR = 1.24, 95% CI 1.12–1.37), decreased age (OR = 0.97, 95% CI 0.96–0.98), single nodule (OR = 1.82, 95% CI 1.27–2.59), and smaller nodule size (OR = 0.78, 95% CI 0.69–0.88) were also identified as independent factors for the diagnosis of DTC.

## 4. Discussion

Using a retrospective study of thyroid nodules, this study demonstrated that the prevalence of DTC was significantly higher in patients with elevated TgAb over 40 IU/mL than those with TgAb under 40 IU/mL. Further analysis revealed that TgAb ≥ 40 IU/mL might be a predictive marker for DTC independent of other potentially confounding factors such as decreased age, single nodule, and elevated TSH level. To the best of our knowledge, this is the first large retrospective study examining the relationship between ATAs and DTC in Chinese population.

Since Dailey et al. [25] proposed an association between thyroid malignancy and Hashimoto disease, the relationship between these two disorders has long been disputed [14, 17, 26]. In one recent prospective study [20], Azizi et al. suggested that the association between Hashimoto disease and thyroid cancer is antibody specific. In agreement with it, we observed that elevated TgAb concentration is associated with high prevalence of DTC. Besides, in our study, a positive correlation between DTC and high serum TPOAb levels was also observed. However, multivariate analyses showed that this association may be ascribed to other potential factors such as TSH, or elevated TgAb.

The link between elevated TgAb levels and DTC incidence rate remains unclear. However, in 1996, a universal salt iodization (USI) program was initiated in China. Since then, our previous epidemiological surveys identified an increased prevalence of DTC in regions with excess iodine intake compared to regions with iodine deficiency, and almost all the subjects with DTC had PTC. In addition, the incidence of positive TgAb was higher in regions with excess iodine intake [23, 27]. These data suggest that, since the initiation of the USI program, regions with adequate iodine intake have also been exposed to greater amounts of iodine. Historically, Shenyang is a region characterized by sufficient iodine intake. Notably, all the patients from Shenyang were enrolled in our study cohort after the implementation of the USI program and would therefore also have had increased iodine intake. Interestingly, the high proportion of PTC for this region (96.6%, *n* = 229) was consistent with a generally accepted fact that DTC was mainly PTC in regions with excess iodine intake [27]. Thus, we speculate that increased iodine intake is the positive link between elevated TgAb levels and DTC incidence rate.

The influence of previous thyroid autoimmunity on the outcome of patients with DTC remains controversial. Souza et al. [28] reported that preexisting thyroid autoimmunity or ATAs exerted a protective effect on the outcome of DTC. Specifically, patients with ATAs and a previous history of thyroid autoimmunity had a fewer metastases and reduced recurrence compared to those without a history of autoimmunity. Recently, a large retrospective survey further supported the beneficial effect of thyroid autoimmunity on DTC [29]. On the contrary, we did not observe a correlation between lymph node metastasis or advanced cancer stages and elevated TgAb or TPOAb levels. Interestingly, however, we did observe a significant increase in extrathyroidal invasion among patients with elevated TgAb. Consistent with our findings, Kim et al. [30] reported an increased recurrence rate in patients with positive TgAb (18% versus 1%, *P* < 0.001), and positive TgAb was independently associated with extrathyroidal extension (HR = 6.05; 95% CI, 1.4–26.5; *P* = 0.02). Moreover, Seo et al. [31] reported that tumour recurrence was significantly higher in patients with elevated TgAb level over 140 U/mL (four times of the normal range) than group with TgAb level under 140 U/mL. And more interestingly, one recent study [32] found that the DTC patients with TgAb epitope restriction similar to Hashimoto disease had higher recurrent/persistent rates (81% versus 17%, *P* < 0.001) and remarkably higher TgAb concentration (887.0 versus 82.0 kIU/L; *P* < 0.001). These studies revealed a nonprotective role of TgAb/autoimmune thyroiditis on DTC.

Serum TSH levels have also been associated with DTC. In this study, high TSH levels were related to elevated TgAb (*P* = 0.018) and TPOAb (*P* = 0.003). Furthermore, logistic regression analysis revealed that elevated TSH levels might be an independent risk factor for DTC, despite the evaluation based on the overall TSH level. This data is consistent with previous reports [3, 15, 20].

Many clinical risk factors, such as male gender, younger age, large nodule size, and single nodule, have long been recognized as predictors of malignancy in patients with thyroid nodules [33–35]. Similar to these studies, decreased age and single nodule were also correlated with DTC in our work. However, we did not observe a difference in DTC with regard to gender. This difference may be related to the following reasons: for one thing, subjects in this study are the Chinese population, which may have had its own characteristic since a USI program was initiated; for another, the inclusion criteria in this study may be different from the other studies. Interestingly, smaller nodule size was associated with DTC in our study, which is more consistent with the work of Kim et al. [15]. Because smaller nodule size is more frequent in patients with PTC malignancy [36, 37], these contrasting data may have resulted from a predominant proportion of patients diagnosed with PTC (96.6%, *n* = 229) in our study.

Although our study further supports a correlation between ATAs/thyroid autoimmunity and DTC, some limitations do exist. First, as a retrospective survey, all data based on surgical specimens may be subject to selection bias. However, FNAB was not routinely performed in diagnostic workup before surgical procedure. Second, the relationship between infiltrating lymphocytes, TgAb levels, and DTC was not analyzed because this information was not available in the pathology record.

In conclusion, our data revealed that the presence of elevated TgAb correlated with an increased risk for DTC. High serum TgAb levels may serve as a predictive marker for DTC independent of TSH levels. In addition, we did observe a significant increase in extrathyroidal invasion among patients with elevated TgAb. Together with other reports, our data suggests that elevated TgAb along with other risk factors, such as decreased age, single nodule, and elevated TSH level, may provide useful information for the diagnosis and prognosis of DTC.

## Figures and Tables

**Table 1:** Clinical characteristics of the final study cohort.

	DTC	Benign	*P* value
Number (*n*)	237	1,401	
Gender (*n*, %)			0.209
Male (*n* = 355)	44 (18.6)	311 (22.2)	
Female (*n* = 1,283)	193 (81.4)	1,090 (77.8)	
Mean age (yr)	43.4 ± 12.9	48.4 ± 11.1	<0.001
Median TSH (mIU/L)^a^	1.53 (0.88–2.42)	1.05 (0.64–1.78)	<0.001
Mean nodule size (cm)	2.7 ± 1.9	3.3 ± 1.6	<0.001
Nodule type (*n*, %)			<0.001
Solitary (*n* = 321)	80 (33.8)	241 (17.2)	
Multiple (*n* = 1,317)	157 (66.2)	1,160 (82.8)	
TgAb ≥ 40 IU/mL (*n* = 233)	56 (23.6)	177 (12.6)	<0.001
TPOAb ≥ 50 IU/mL (*n* = 136)	30 (12.7)	106 (7.6)	0.009

^a^TSH levels are presented as median (25th–75th empirical percentiles).

DTC: differentiated thyroid cancer.

**(a):** 

	O1 (TPOAb < 50 IU/mL)	O2 (TPOAb ≥ 50 IU/mL)	*P* value
Number (*n*)	207	30	
Gender (*n*, %)			0.197
Male (*n* = 44)	41 (19.8)	3 (10)	
Female (*n* = 193)	166 (80.2)	27 (90)	
Mean age (yr)	44.15 ± 12.64	38.53 ± 13.50	0.025
Median TSH (mIU/L)^a^	1.46 (0.84–2.24)	2.09 (1.38–3.20)	0.003
Mean nodule size (cm)	2.71 ± 0.14	3.14 ± 0.55	0.95
Nodule type (*n*, %)			0.718
Solitary (*n* = 80)	69 (33.3)	11 (36.7)	
Multiple (*n* = 157)	138 (66.7)	19 (63.3)	
Lymph node metastasis	60 (29)	13 (43.3)	0.112
Extrathyroidal invasion	42 (20.3)	6 (20)	0.971
Advanced stages (III, IV)	75 (36.2)	15 (50)	0.146

^a^TSH levels are presented as median (25th–75th empirical percentiles).

DTC: differentiated thyroid cancer.

ATAs: thyroid autoantibodies.

**(b):** 

	G1 (TgAb < 40 IU/mL)	G2 (TgAb ≥ 40 IU/mL)	*P* value
Number (*n*)	181	56	
Gender (*n*, %)			0.034
Male (*n* = 44)	39 (21.5)	5 (9.9)	
Female (*n* = 193)	142 (78.5)	51 (90.1)	
Mean age (yr)	44.13 ± 12.20	41.23 ± 14.67	0.184
Median TSH (mIU/L)^a^	1.44 (0.77–2.30)	1.72 (1.28–2.57)	0.018
Mean nodule size (cm)	2.49 ± 0.14	3.02 ± 0.31	0.07
Nodule type (*n*, %)			0.207
Solitary (*n* = 80)	65 (35.9)	15 (26.8)	
Multiple (*n* = 157)	116 (64.1)	41 (73.2)	
Lymph node metastasis	52 (28.7)	21 (37.5)	0.214
Extrathyroidal invasion	31 (17.1)	17 (30.4)	0.031
Advanced stages (III, IV)	66 (36.5)	24 (42.9)	0.389

^a^TSH levels are presented as median (25th–75th empirical percentiles).

DTC: differentiated thyroid cancer.

ATAs: thyroid autoantibodies.

**Table 3:** Multivariate logistic regression analysis predicting DTC.

	Odds ratio (OR)	*P* value
	(95% confidence interval)	
Age (yr)	0.97 (0.96–0.98)	<0.001
Sex (male)	1.12 (0.75–1.67)	0.574
Solitary nodule	1.82 (1.27–2.59)	0.001
Size	0.78 (0.69–0.88)	<0.001
TSH	1.24 (1.12–1.37)	<0.001
TgAb ≥ 40 IU/mL^a^	2.10 (1.40–3.15)	<0.001
TPOAb ≥ 50 IU/mL^b^	1.20 (0.71–2.04)	0.499

Serum TgAb and serum TPOAb concentrations were analyzed as categorical variables.

^a^Compared with TgAb <40 IU/mL group.

^b^Compared with TPOAb <50 IU/mL group.

DTC: differentiated thyroid cancer.
